# Transcriptional profiling of gastric epithelial cells infected with wild type or arginase-deficient *Helicobacter pylori*

**DOI:** 10.1186/1471-2180-12-175

**Published:** 2012-08-13

**Authors:** Songhee H Kim, Rosa A Sierra, David J McGee, Jovanny Zabaleta

**Affiliations:** 1 Department of Microbiology and Immunology, Louisiana State University Health Sciences Center-Shreveport, 1501 Kings Highway, Shreveport, LA, 71130, USA; 2 Department of Pediatrics and Stanley S. Scott Cancer Center, Louisiana State University Health Sciences Center, New Orleans, LA, 70112, USA

**Keywords:** Arginase, *Helicobacter pylori*, Interleukin-8

## Abstract

**Background:**

*Helicobacter pylori* causes acute and chronic gastric inflammation induced by proinflammatory cytokines and chemokines secreted by cells of the gastric mucosa, including gastric epithelial cells. Previous studies have demonstrated that the bacterial arginase, RocF, is involved in inhibiting T cell proliferation and CD3ζ expression, suggesting that arginase could be involved in a more general dampening of the immune response, perhaps by down-regulation of certain pro-inflammatory mediators.

**Results:**

Global transcriptome analysis was performed on AGS gastric epithelial cells infected for 16 hours with a wild type *Helicobacter pylori* strain 26695, an arginase mutant (*rocF-*) or a *rocF*^*+*^ complemented strain. *H. pylori* infection triggered altered host gene expression in genes involved in cell movement, death/growth/proliferation, and cellular function and maintenance. While the wild type strain stimulates host inflammatory pathways, the *rocF-* mutant induced significantly more expression of *IL-8*. The results of the microarray were verified using real-time PCR, and the differential levels of protein expression were confirmed by ELISA and Bioplex analysis. MIP-1B was also significantly secreted by AGS cells after *H. pylori rocF-* mutant infection, as determined by Bioplex. Even though not explored in this manuscript, the impact that the results presented here may have on the development of gastritis, warrant further research to understand the underlying mechanisms of the relationship between *H. pylori* RocF and IL-8 induction.

**Conclusions:**

We conclude that *H. pylori* arginase modulates multiple host signaling and metabolic pathways of infected gastric epithelial cells. Arginase may play a critical role in anti-inflammatory host responses that could contribute to the ability of *H. pylori* to establish chronic infections.

## Background

*Helicobacter pylori* (*H. pylori*) causes a spectrum of gastric diseases ranging from mild to severe gastritis and peptic ulcers to gastric cancer [1]. During early stages of infection, H. pylori adheres to the gastric epithelial cells in the gastric pit, leading to induction of chemokines and cytokines. These proinflammatory mediators induce the infiltration of neutrophils and lymphocytes. Despite this immune response, the infection is not cleared [2] and patients can remain chronically-infected for decades if not treated. This chronicity suggests the bacterium has evolved strategies to persist in the gastric mucosa despite strong immune responses, indicating that *H. pylori*, in addition to inducing factors to promote inflammation, may also have factors to dampen the host immune responses.

Several *H. pylori* factors have been associated with virulence including the vacuolating cytotoxin (VacA), the product of the cytotoxin-associated gene (CagA) and the *H. pylori* urease [3-9]. However, the mechanisms of pathogenesis caused by other *H. pylori* factors are only beginning to be understood.

*H. pylori* arginase [EC 3.5.31, RocF] hydrolyzes arginine to ornithine and urea, the latter of which may serve as an endogenous substrate for the powerful *H. pylori* urease enzyme, to generate carbon dioxide and ammonia. The *H. pylori* RocF is associated with the inner cell membrane and uses cobalt as cofactor, as opposed to mammalian arginases which use manganese [10-12]. Interestingly, arginase activity has an acidic pH optimum and increases the resistance of *H. pylori* to acid in an arginine-dependent fashion [11]. Moreover, since the *rocF-* mutant is unable to hydrolyze and consume arginine [13,14], extracellular arginine levels are readily available for macrophages to produce nitric oxide (NO) to kill the bacteria [15].

Both in a tissue culture system and from peripheral blood from human volunteers, it was shown that, in contrast with wild type *H. pylori*, the *rocF-* mutant promotes T cell proliferation and expression of the important T cell surface signaling molecule, CD3ζ [16]. Thus, arginase is involved in dampening the innate (acid, NO) and adaptive (T cell) immune responses, but the specific mechanisms are not entirely understood. *H. pylori* arginase in gastric epithelial cell response is unknown. We therefore sought to determine the impact of *H. pylori rocF-* on epithelial cell transcription and cytokine/chemokine profiles using Illumina gene chip analysis, real-time PCR, ELISA and Bioplex analysis.

## Results

### Differential gene expression profile between *H. pylori* 26695 wild type and *rocF-* mutant strains

**Figure 1:**
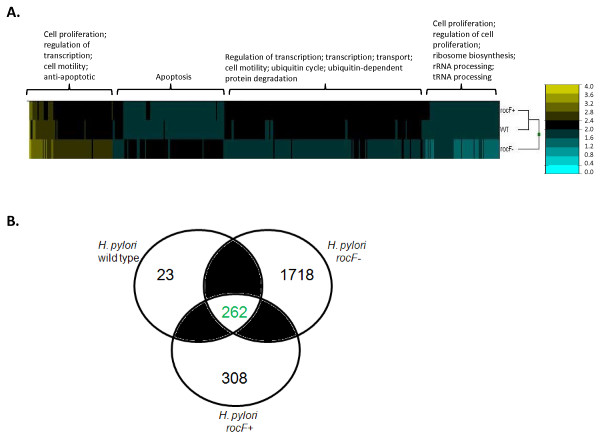
**Differential gene expression profiles of AGS gastric epithelial cells infected with WT,*rocF-*and the*rocF***^**+**^**complemented*H. pylori*strains. A**. Representative portion of the Log10 ratio between the net expression values between the infected and the non-infected cells, as described in Materials and Methods. The analysis was done using four replicates of each treatment. The marked areas above the heat map show genes associated with different cellular functions. **B**. Venn diagram showing the number of genes affected (up- and down-regulated) by the infection of AGS cells with the WT, *rocF*-, or *rocF* + strains of *H. pylori*. The green number (262) indicates the number of genes that are common to all treatments; the black numbers indicate unique genes in each treatment; the total shaded area represent 583 genes that are neither common nor unique (similar genes).

**Figure 2:**
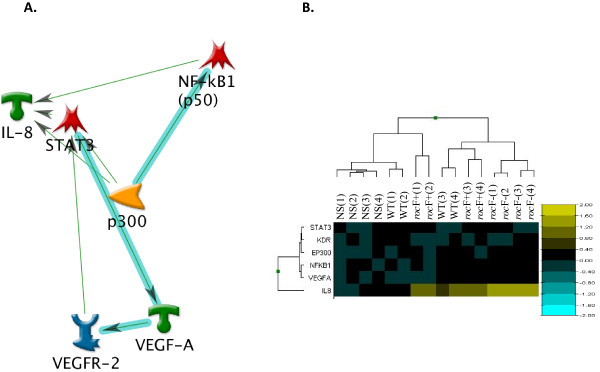
**Network interactions in AGS cells infected with*H. pylori*. A**. Expanded central node of a network (*RelA* (p65), *NFkB*, c-*IAP2*, *NFkBIA*, and *MUC1*) generated using the net gene expression values of the different *H. pylori* infections of the AGS cells. Green arrow = positive regulation; green icons represent receptor ligands (*IL-8*, *VEGFA*); red icons represent transcription factors (*NFKB1*, *STAT3*); yellow icon represent generic enzyme (*p300*). Thicker arrows indicate stronger association. **B**. Heatmap showing the similarity of the different replicates, using the Log10 ratio of the expression values, as explained in Figure 1. Both Figures were generated using data from four replicate independent experiments.

Gastric adenocarcinoma epithelial cell line AGS has been extensively studied and reviewed as a valid *in vitro* model for *H. pylori* interactions [17]. *H. pylori* arginase, encoded by *rocF*, plays an important role in both innate and adaptive immunity [15,16], but nothing is known about the gastric epithelial response. This question was addressed by transcriptome analysis of AGS cells infected by wild type, the *rocF-* mutant, and *rocF*^+^ complemented *H. pylori* strains. The log10 transformed data of the net intensity signal, using non-infected cells (NS) as reference, was used to generate a heat-map of gene expression profiles of the different *H. pylori* treatments in AGS cells. As seen in Figure 1A, the expression profile of both WT and the complemented *rocF*^+^ was very similar. In contrast, the gene profile obtained in response to the infection with the *H. pylori* arginase mutant (*rocF*-) was completely different to the profiles generated by the other two strains as evidenced by the localization of the *rocF*- strain in a separate branch of the dendrogram. Interestingly, a set of genes associated with pro-apoptotic and anti-apoptotic pathways were differentially expressed in the *rocF-* mutant as compared to the wild type or *rocF*^+^ strains (Figure 1A). In addition, infection with the *rocF-* mutant affected the expression of more genes than WT while the number of genes was similar in both number and intensity between the WT and the complemented bacteria. Using Metacore software analysis(Thomson Reuters, Philadelphia, PA), we found that while 262 genes were common to the infection with all three *H. pylori* strains, infection with *rocF*- resulted in modulation of 2,563 genes of which 1,718 were uniquely induced by this strain (Figure 1). In contrast, compared to *rocF*-, infection with either the WT or the *rocF*^+^ induced a lower number of genes (868 and 1153, respectively) of which only 23 were uniquely induced by the WT strain and 308 by the *rocF*^+^ (Figure 1B). All three combined shaded areas represent 583 “similar” genes, those that are not “unique” to each treatment, or “common” to the three conditions, but are similar to any pair of treatments. To understand how these genes interact we generated networks and pathways maps using the MetaCore software. The network with the maximum G-score (127.02, based on the number of interactions), with a *p* = 2.1 x 10^-16^ (*RelA, NFκB, c-IAP2, NFKBIA, MUC1*) was assembled and showed a central core formed by the *NFκB* family. This central core was further expanded to highlight the most relevant genes (those with stronger associations) and this revealed a set of genes associated with inflammatory responses, including *IL-8NFκB*, and *STATs* (Figure 2A). It is noteworthy that, based on the network, *IL-8* is one of the most modulated genes in this central core, with interactions with several other genes, including *NFKBNFKB1STAT3*, and the histone acetyl-transferase *p300* (*EP300*), the latter functioning as an *IL-8* activator either directly or indirectly through the activation of other genes involved in *IL-8* transcription (Figure 2A). Figure 2B shows the similarity of the replicates (numbered in parenthesis) using the net intensity of the transcripts shown in Figure 2A. As observed, the dendrogram pattern shows that WT and *roc*F + *H. pylori* are similar as they mix together, while the *rocF*- segregates in a separate branch of the dendrogram, showing different patterns of expression. Pathway maps analysis revealed the importance of the immune system in the *H. pylori* infection. The map showing the highest significance was associated with immune response (*p* value 1.018 x 10^-5^) and involved many of the genes present in the network, including *IL6IL-8NFKBAP-1JUN*, and *IL1B* (data not shown). Even though *IL-8* was the most modulated gene from our microarray analysis, it was not the only one. Table 1 shows the raw and the net expression signals of the 10 most up- and the 10 most down-regulated genes in AGS cells infected with the different strains of *H. pylori*. Based on the direct analysis of the gene list, and those obtained from networks and pathways analysis, and very especially on the role of IL-8 in the induction of inflammatory responses, we focused our efforts on confirming the effects of the infection on *IL-8* production.

**Table 1:** **Ten most up- and 10 most down-regulated genes in AGS cells in response to the infection with the different strains of*H. pylori***

			**Raw Signal**			Net Signal*		
		***H. pylori*strain**			***H. pylori*strain**			
**TargetID**	**NS**	**WT**	***rocF*-**	***rocF***^**+**^	**WT**	***rocF*-**	***rocF***^**+**^	
*IL8*	130.5	531.8	4021.7	1276.8	401.3	3891.2	1146.3
*S100A3*	143.6	298.2	1488.3	463	154.6	1344.7	319.4
*KRT17*	1115.3	2555.1	11710.4	7149.9	1439.8	10595.1	6034.6
*LCP1*	214.4	351.2	1585.8	568.8	136.8	1371.4	354.4
*SERPINB2*	116.2	129.1	547.4	235.8	12.9	431.2	119.6
*RND1*	113.6	171.3	576	195.7	57.7	462.4	82.1
*ACTG2*	402.8	417.7	1388.5	723.4	14.9	985.7	320.6
*SPOCD1*	170.4	250.4	748	321.4	80	577.6	151
*RASD1*	157.5	192.8	563.6	269.5	35.3	406.1	112
*PLAUR*	450.2	1714	4856.2	1649.2	1263.8	4406	1199
*RPP40*	2648	1581.3	591.7	2117.1	−1066.7	−2056.3	−530.9
*RRS1*	596.6	397.5	148.2	477.9	−199.1	−448.4	−118.7
*CABC1*	1038.4	698.2	254.1	652.8	−340.2	−784.3	−385.6
*ZNF239*	591.8	389.4	139.8	409.2	−202.4	−452	−182.6
*SLC1A3*	1269.7	1028.9	364.7	875.9	−240.8	−905	−393.8
*SOX2*	652.5	373.5	126.3	389.7	−279	−526.2	−262.8
*LOC91461*	830.4	527.4	160.9	606.7	−303	−669.5	−223.7
*FGD3*	654.5	384.4	115	262.7	−270.1	−539.5	−391.8
*ATF7IP2*	1059	662.3	185.1	665.7	−396.7	−873.9	−393.3
*DKK1*	5514.2	2808.6	264.6	2722.3	−2705.6	−5249.6	−2791.9

*Net signal is obtained by subtracting the raw value from the values obtained in *H. pylori*-infected AGS cells. NS, Non-infected AGS cells.

### The *rocF- H. pylori* mutant induces more *IL-8* in gastric epithelial cells than wild type *H. pylori*

**Figure 3:**
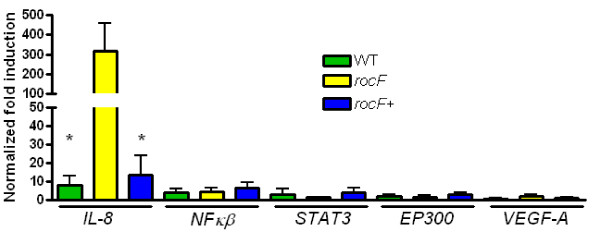
**Infection with the*H. pylori*26695*rocF-*mutant induces significantly higher levels of*IL-8*than its wild type or*rocF***^**+**^**counterparts.** Fold induction of genes depicted in Figure 2, performed as explained in Materials and Methods using GAPDH as housekeeping gene and one internal calibrator. * *p* < 0.0001, as compared to the induction in response to the infection with *H. pylori rocF*-. Values represent the average expression ± SEM of three independent replicates.

We used real-time PCR to confirm the expression of the genes shown in Figure 2. For this, we obtained the fold induction of each gene (ΔΔCt) of the expression with *GAPDH* as housekeeping and normalizing with an internal calibrator. The fold induction at 0 h was subtracted and the signal obtained in the NS used to determine the ratio of the induction of each gene in WT, *rocF*- and *rocF*^+^ infected AGS cells. As seen in Figure 3, infection with the *H. pylori rocF-* mutant induced 40 and 23 times more *IL-8* than the *H. pylori* WT or the *rocF*^+^ complemented strain, respectively (*p* < 0.0001). No significant difference was found in the fold induction of the other genes (Figure 3). The data suggest that the *H. pylori* arginase may act as an important modulator of inflammatory responses through the control of IL-8 transcription in gastric epithelial cells.

**Figure 4:**
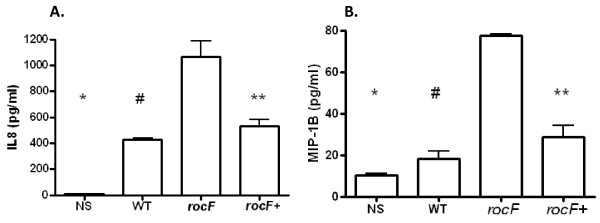
**The*H. pylori rocF-*mutant induces more IL-8 and MIP-1βin AGS cells than wild type*H. pylori,*as determined by Bioplex.** Supernatants from *H. pylori* infected-AGS cells were collected and used to determine the concentration of IL-8 and MIP-1β (pg/ml) **A**. Levels of IL-8; one-way ANOVA p < 0.0001; *p = 0.0001 (*rocF-* vs NS); #*p* = 0.0249 (*rocF-* vs WT); **p = 0.044 (*rocF-* vs *rocF*^+^); **B**. Levels of MIP-1B; one-way ANOVA *p* < 0.0001; *p < 0.0001 (*rocF-* vs NS); #*p* < 0.0001 (*rocF-* vs WT); *p* = 0.0001 (*rocF-* vs *rocF*^*+*^). Values in both Figures represent the average signal ± SEM of four independent replicates.

**Figure 5:**
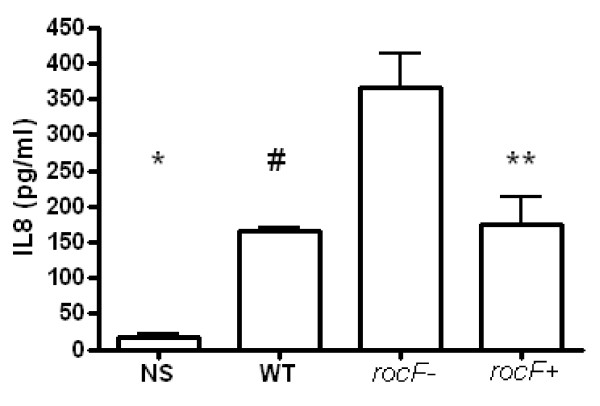
**The*rocF*mutant of*H. pylori*induces more IL-8 in AGS cells compared with wild type*H. pylori,*as determined byELISAanalysis.** Please see legend on Figure 4 for IL-8. One-way ANOVA *p* = 0.0002; *p = 0.0003 (*rocF-* vs NS); #*p* = 0.045 (*rocF-* vs WT); **p = 0.0185 (*rocF-* vs *rocF*^+^). Values represent the average signal ± SEM of four independent replicates.

Due to the biological importance of IL-8 and because the microarray suggested wider and stronger cytokine inductions by *H. pylori* 26695 *rocF-* mutant than the wild type and the complemented bacteria at the transcriptional level, Bio-Plex analysis was further pursued to simultaneously examine 27 different human cytokines and chemokines (Human Cytokine Assay Group 1 platform). Fourteen cytokines and growth factors were induced by at least one of the *H. pylori* strains. IL-8 was the most abundantly expressed cytokine/chemokine, especially by the AGS cells infected with the *H. pylori rocF-* mutant strain (1068 ± 243.8 pg/ml) as compared to the WT (428 ± 13.4) or the complemented isogenic strain (529 ± 73.1) (Figure 4A). From the Bio-plex analysis it was evident that, in addition to IL-8, the *rocF-* bacteria also induced higher levels of MIP-1B, as compared with the other strains (Figure 4B). To confirm the Bio-Plex results we checked the levels of IL-8 by ELISA and found that, indeed, the *H. pylori rocF-* mutant induced more than two times more IL-8 in AGS cells, as compared to the WT or the *rocF* + complemented bacteria (Figure 5).

## Discussion

While it is well-known that *H. pylori* induces inflammation, this inflammatory response is insufficient to clear the organism from the gastric mucosa and the organism overcomes the immune response to cause chronic infections that can last for decades in untreated patients. Paradoxically, *H. pylori* may have both pro-inflammatory as well as anti-inflammatory mechanisms. These opposing forces must operate in such a fashion as to achieve a delicate balance that involves complex interactions between bacterial virulence factors and host innate and adaptive immune system factors.

How does arginase in wild type *H. pylori* act as an anti-inflammatory mediator? While the underlying mechanisms are still not well understood, the depletion of arginine by this enzyme from the extracellular environment may be one factor that triggers altered gene expression in the gastric epithelial cell. Precedence for this idea comes from prior work showing that arginine depletion leads to altered T cell receptor zeta chain expression (CD3ζ) [16]. Another possibility is that the products of arginine hydrolysis, namely, ornithine and urea, could also be playing a role in altering transcriptional responses by the gastric epithelial cells. A third possibility is that the arginase mutant, through disruption of the bacterial metabolic balance of arginine, ornithine, or urea levels, could have altered gene transcriptional profiles leading to modified expression of other bacterial virulence factors that interplay with the host immune system.

A fourth possibility is that the increase in IL-8 production induced by the *H. pylori rocF-* mutant is through altered spermine produced by the AGS cells. Previous reports have shown that *H. pylori* infection induces ornithine decarboxylase (ODC) in macrophages [15,18]. ODC degrades L-ornithine into putrescine and this is later converted into spermidine and finally spermine. Although we did not find differences in the level of mRNA for the spermine synthase (*SMS*), we did find that ornithine decarboxylase (*ODC*) and spermidine synthase mRNA (*SRM*), two key enzymes in the production of spermine, were 20% and 41%, respectively, reduced in the *rocF-*mutant*-*infected AGS cells (data not shown). It has also been shown that spermine can reduce the inflammatory response by post-transcriptional inhibition of the production of pro-inflammatory cytokines, including TNFα, IL6, MIP-1α, and MIP-1β [19], and even though IL-8 was not included in this study, it is possible that it is regulated by spermine as well. Thus, in the interaction of wild type *H. pylori* with AGS cells, spermine levels may be elevated in the AGS cells, leading to a dampening of the chemokine/cytokine pro-inflammatory response. These possibilities await further in depth analyses.

We performed pair-wise comparison of transcriptome on the human adenocarcinoma gastric cell line AGS after infection with 26695 wild type, its isogenic *rocF-* knockout mutant, and a *rocF-* complemented (*rocF*+) *H. pylori* strain, with uninfected AGS cells as a control. The first observation with the microarray analysis was an overall increase in the number of genes that participate in several signaling pathways previously investigated with *H. pylori* infection, notably with *NFKB* and *AP-1* activation and mitogen-activated protein kinase (especially *ERK*s, *JNK*s, *SAPK*s) [20], along with *JUN*-mediated signaling. From this activation cascade, the induction of *IL-8* marked the greatest difference between the *rocF-* mutant *H. pylori* versus either the WT or the *rocF*^*+*^ complemented strain. Our results show a significant increase of mRNA and protein levels of *IL-8* in AGS cells infected with the *rocF-* mutant strain, suggesting that WT bacteria may be able to control the inflammatory infiltration of immune cells by controlling the production of IL-8, which is a potent chemotactic factor for inflammatory cells, especially neutrophils [21-24].

While many *H. pylori* factors have been suggested to stimulate IL-8 expression, including peptidoglycan, LPS, CagA, VacA, PicB, IceA, urease (and even ammonia) [25-28], less is known about bacterial factors involved in suppression of cytokine production, especially in epithelial cells. Mechanisms for immune evasion by *H. pylori* have been demonstrated, including the presence of a less potent LPS and cholesterol glycosylation [29]; however, fewer studies dealt with reduced host cytokine production as an immune suppressive mechanism, including effects on IL-12 [30-32]. While an increased amount of cytokines can result in histologically more intense gastritis [33], the limitation of this cytokine induction could be an advantage to the bacteria so that it can stay under the radar of the immune system. However, due to the complexity of the *H. pylori*-gastric cell interaction, and the complexity of the lesions induced by the infection, it is expected that multiple pathways are activated and the balance of those pathways may determine the presence or the evolution of the gastric lesions.

Efforts to determine the effect of the infection with *H. pylori rocF*- strains in the cellular infiltration of the gastric mucosa are currently underway. To the best of our knowledge, there is only one published work trying to measure the levels of *H. pylori* arginase in gastric biopsies of patients with gastritis and its correlation with disease [34]. That work showed that there is a lot of variability on the levels of *H. pylori* arginase in biopsies but the authors were not able to establish a correlation with the degree of gastritis. The reason for the increased number of genes modulated by the *rocF*- *H. pylori*, when compared to the WT and the *rocF*^+^ bacteria, is not known; however, our results, rather than suggesting the existence of *H. pylori* arginase mutants in human gastric lesions, highlights the importance that this enzyme may have in the interaction of the bacteria with cells in the human gastric mucosa, and through them, with the immune system. Taken together our results suggest that *H. pylori* arginase, by modulating the production of IL-8 may play a significant role in the survival of *H. pylori* in the gastric environment. By preventing an over-zealous immune response, *H. pylori* can achieve its chronicity through the production of arginase and probably other bacterial factors that contribute to the overall global success of this important and highly-adapted gastric human pathogen.

## Conclusion

Our results highlight the importance of *H. pylori* arginase in the modulation of inflammatory responses. Since IL-8 is pivotal for the infiltration of inflammatory cells into the gastric mucosa, *H. pylori* arginase may be involved in reducing the tissue damage associated with the evolution of the gastric lesions through the modulation of multiple pathways on the gastric epithelial cells.

## Methods

### Bacterial growth conditions

*H. pylori* 26695 strains (wild type [WT], *rocF-* mutant, and the *rocF-* mutant chromosomally-complemented with wild type 26695 *rocF-* (*rocF-*26695-MLB0004*,* hereafter referred to as *rocF+*) were described previously [13]. All strains were passaged every 2–3 days on Campylobacter blood agar (CBA) plates at 37°C with 85% N_2_, 5% O_2_, and 10% CO_2_. Prior to coculture experiments, *H. pylori* cells were grown in Ham’s F-12 with heat-inactivated 1% (v/v) fetal bovine serum (FBS) [35]. *H. pylori* growth was monitored by ATP level using Cell Titer-Glo® cell viability assay kit (Promega, NY, USA), as validated previously [36] and by plating for colony forming units. Comparable number of viable bacteria was assured in each experiment.

### Tissue culture and co-culture

AGS gastric epithelial cells (ATCC CRL-1739, Rockville, MD) were maintained in F-12 with heat-inactivated 10% FBS at 37°C in an atmosphere of 5% CO_2_. For the experiments, 1 x 10^6^ AGS cells were seeded into 6-well plates containing 2 ml fresh F-12 supplemented with 3% heat-inactivated FBS and cultured for 8 hours. The media was replaced with 2 ml fresh F-12 containing 3% heat-inactivated FBS before inoculation of the different *H. pylori* at a multiplicity of infection (MOI) of 20. The infected cells were cultured for additional 16 h after which the media was collected and stored for ELISA and BioPlex analyses and the RNA extracted for microarray and real-time PCR studies.

### RNA extraction and microarray

To extract the RNA from the AGS cells, coculture supernatants were removed by aspiration and 1 ml of TRIZOL (Invitrogen, Carlsbad, CA) was added immediately to each well. RNA was extracted as recommended by the manufacturer and was stored at −80°C until further use. RNA was dissolved in DNase/RNase-free water, quantified by NanoDrop (Fisher Scientific) and set at a concentration of ~1.0 μg/μl. The quality of the RNA was confirmed by Agilent 2100 Bioanalyzer (Agilent Technologies, Palo Alto, CA). Each experiment was repeated four times. Two hundred ng of RNA were used to make biotinylated cRNA using the Illumina TotalPrep RNA Amplification Kit (Ambion, Austin, TX), and hybridized to the Illumina chips for 14 hours at 58°C. After washing and staining, the arrays were scanned with the BeadArray Reader (Illumina Inc.) and analyzed with the GenomeStudio software (Illumina).

### Microarray data analysis

After subtracting the background, the samples were normalized assuming a similar distribution of transcript abundance in all the samples [37]. The net expression level was obtained by subtracting the intensity obtained on each treatment (including non-treated cells) from the intensity at 0 h (prior to seeding the cells into the plate). Then, the gene levels on the infected cells were compared against the levels on the non-infected cells setting the *p* value for the difference at <0.05. Scatter plots comparing the non-infected cells against each one of the other treatments (AGS + WT, AGS + *rocF-*, AGS + *rocF*^+^) were used to select only those genes with > 3 fold difference (up or down-regulated) as compared with the non-infected cells, and *p* values less than 0.05 (*p* < 0.05). In addition, the Log_10_ of the ratio between the normalized intensity in the infected cells and the normalized intensity in the non-infected cells was determined and used to generate heat maps.

### Quantitative real-time PCR

For real-time PCR (qPCR), total RNA extracts were DNase treated and reverse-transcribed with SuperScriptase III (Invitrogen) with random hexamers. TaqMan pre-designed arrays were used to check the levels of mRNA expression of IL-8, using cyclophilin A as housekeeping gene, and following the vendor’s recommendations (Applied Biosystems, Foster City, CA). The 5 μl reaction was subjected to two minutes at 50°C, 10 minutes at 95°C and finally 40 cycles at 95°C for 15 seconds and 60°C for one minute in a 7900HT real-time PCR machine (Applied Biosystems). The delta Ct’s (ΔCt and ΔΔCt) and fold induction of IL-8 were determined using an internal control as calibrator.

### BioPlex and enzyme-linked immunosorbent essay (ELISA)

Culture supernatants were collected at indicated time points and clarified of bacteria and host cells by centrifugation (3,000 x *g* for 5 min and then 16,000 x *g* for 10 min, 4°C). Manufacturer’s manuals were followed for BioPlex (Bio-Rad, Inc) and human IL-8 ELISA assay (BD OptEIA^TM^, BD Bioscience). For Bio-Plex analysis, 2 μl of anti-cytokine conjugated beads were added to each well, followed by diluted culture supernatants. After 30 min incubation, samples were washed three times with Bio-Plex wash buffer, and then 25 μl of detection antibody solution was added and incubated for another 30 min. Streptavidin-phycoerythrin (1X; 50 μl) was added to each well and then washed. For hIL-8 ELISA, duplicate measurements were done for four separate experiments. Samples were read at 450 nm on an ELISA reader (Bio-Rad), of which lowest detection limit was 0.8 pg/ml (BD OptEIA^TM^, BD Bioscience).

### Functional analysis and network generation

Online computational tools of Metacore (Thomson Reuters, Philadelphia, PA) were used to identify annotated networks of interacting genes, pathways and associated biological functions among genes profiled from the microarray analysis, using more than 700 canonical maps and pathways which are continuously being updated (http://www.genego.com). The networks generated were ranked and built according to G-scores and *p* values.

### Statistical analysis

All data in each experiment of ELISA and real time PCR are presented as mean ± SEM of three or four different experiments. To check for any difference between the several treatments we did a one-way ANOVA analysis. To determine differences between specific treatments we did a two-tailed unpaired *t*-test.

## Authors’ contributions

SHK and RAS conducted all the experiments described in the manuscript; DJM and JZ designed the study, provided support and helped with the experiments, and co-wrote the manuscript. All authors read and approved the final manuscript.
